# Enhancing personalized suicide risk prediction for VA patients by integrating discrete natural language processing models

**DOI:** 10.1038/s41398-026-03940-8

**Published:** 2026-03-20

**Authors:** Monica Dimambro, Joshua Levy, Jiang Gui, Matan Goldberg, Brian Shiner, Maxwell Levis

**Affiliations:** 1 White River Junction VA Medical Center, White River Junction, VT USA; 2 Pathology and Computational Biomedicine, Cedars Sinai Medical Center, Los Angeles, CA USA; 3 Geisel School of Medicine at Dartmouth, Hanover, NH USA; 4 Jerusalem College of Technology, Jerusalem, IL Israel; 5 National Center for PTSD Executive Division, White River Junction, VT USA

**Keywords:** Human behaviour, Psychology

## Abstract

To improve the identification of Veterans at risk for suicide, the U.S. Department of Veterans Affairs (VA) developed REACH-VET, a suicide risk classification metric. Our previous work demonstrated that incorporating natural language processing (NLP) and developing targeted models for distinct suicide risk-tiers could enhance REACH-VET’s predictive accuracy. This study evaluates the benefits of two NLP methods and compares their predictive performance across risk-tiers. We created a sample of VA patients who either died by suicide in 2017–2018 (cases) or remained alive during that period (controls), stratified by suicide risk (high, moderate, low). We analyzed unstructured electronic health record (EHR) notes using two NLP models: 1) theory-based, closed-vocabulary “semantic” methods, and 2) data-driven, open-vocabulary “count” methods. We then developed eXtreme Gradient Boosting (XGBoost) classification models using semantic, count, and hybrid (count and semantic) variables and calculated area under the receiver operating characteristic curve to assess predictive accuracy. Generally, classification models using semantic variables performed worse than count or hybrid variables. The highest added benefit was seen for low- and moderate-risk patients who achieved incremental improvements in performance over and above leading predictive benchmarks. By using different NLP techniques on unstructured EHR data, our approach improves predictive accuracy for lower-risk patients, expanding suicide prediction for patient populations who are poorly understood and frequently underserved.

## Introduction

Veterans have a substantially elevated suicide risk burden when compared to their civilian counterparts [1–3]. To better identify, track, and treat Veterans at risk for suicide, the United States Department of Veterans Affairs (VA) has prioritized the development of targeted suicide risk prediction models [4]. These efforts focus primarily on identifying patients at the highest predicted suicide risk-tier, a subgroup which includes a disproportionate number of annual suicide deaths [5, 6].

To improve suicide risk classification, leading healthcare networks including the VA, Mayo Clinic, and Kaiser Permanente [4, 7, 8] have turned to machine learning to quantify personalized suicide risk [9, 10]. The VA, an early adopter of leveraging machine learning to individualize care, developed Recovery Engagement and Coordination for Health–Veterans Enhanced Treatment (REACH-VET), a suicide risk prediction model which automatically calculates risk of suicide based on structured electronic health record (EHR) variables, including suicidal ideation, mental health diagnoses, and service utilization [4]. Structured data in this context refers to data that are already tabular, like a list of ages or a binary indicator of having or not having a particular diagnosis. REACH-VET uses multivariate logistic regression with established parameter weights to predict a patient’s risk of suicide in the subsequent 12-months. That value is then converted to a percentile based on the facility’s REACH-VET suicide risk probability distribution.

In contrast to structured data, unstructured data are data that are not yet processed for use in mathematical modelling, such as free text found in a doctor’s note. Medical fields, including oncology [11], emergency medicine [12], and intensive and critical care [13, 14], have successfully used Natural Language Processing (NLP), an umbrella term for computational methods of transforming qualitative data such as free text into quantitative formats [15]. This method allows researchers to incorporate unstructured data alongside structured data to improve predictive risk models [16]. Although REACH-VET is the VA’s most sophisticated suicide prediction method [17], research suggests its predictive accuracy could be improved by shifting towards a more personalized risk framework by expanding beyond its current set of structured predictors [18]. This targeted risk approach aligns with the increasing focus on personalized or “precision” care [19], a methodology that centers on patients’ individual characteristics.

Our prior work has prioritized enhancing personalized suicide risk prediction in the following two ways: Firstly, to better focus on patient differences, we developed novel methods of extracting personalized risk variables from unstructured EHR data [20–22]. To do this, we used NLP to derive psychosocial risk variables from unstructured EHR. Our findings suggested that these personalized variables aid risk prediction efforts, significantly enhancing models like REACH-VET. Secondly, we investigated the unique characteristics of suicide decedents across the range of predicted suicide risk [23]. This work developed a pragmatic way to differentiate these populations into high-, moderate-, and low-risk-tier subgroups of suicide decedents and described demographic and service use differences across these groups.

Our previous NLP investigations used both dictionary-based semantic analysis, an approach that uses groups of words that are theoretically associated with specific concepts [24], and count-based methods, measuring the frequency and distribution of given words within a corpus [20]. These methods are referenced as using “closed vocabularies” and “open vocabularies”, respectively [25], as the first evaluates a specific subset of pre-specified words, while the second evaluates statistical patterns among all corpus words. Dictionary-based sentiment analysis, such as Sentiment Analysis and Cognition Engine (SÉANCE) [26], group together multiple terms, allowing for measurement of defined psychosocial variables. Count Vectorization [27], a method that converts text into matrices of token counts, where each column represents a token and each row represents a document, allows data patterns to independently emerge without pre-determined theoretical assumptions. While these methods have been shown to offer contrasting benefits, available research suggests synergistic effects of leveraging both approaches together [25]. Although this synergy has been achieved in other domains [28], the combined approach has yet to be applied to suicide risk modeling. The primary aim of this study is to demonstrate a novel way to synergistically integrate these NLP methods in the development of a tree-based personalized classification model that optimally predicts suicide risk across risk groups.

## Materials and methods

### Sample selection

To construct our cohort, we linked data from the VA Corporate Data Warehouse (CDW) [29] Electronic Health Records (EHR) with mortality data from the VA-Department of Defense Mortality Data Repository (MDR) [30] to identify individuals who died by suicide and had at least one encounter with VA healthcare in either 2017 or 2018 (*n* = 4584 cases). Following recommendations regarding rare events case-control studies [31], as well as our prior matching methodology [20], each case was paired with 5 controls. The VA Office of Mental Health and Suicide Prevention assisted us in selecting controls who received care at the same VA facility during the same timeframe and, at the time of case death, were alive and shared the same REACH-VET risk percentile at the time of the case’s death (*n* = 22,657 controls). To pursue a stratified approach, we classified each patient into “high”, “moderate”, or “low” risk-tiers based on previously established REACH-VET percentile cut points, where “high” is a 1, “moderate” is 2-24, and “low” is 25-100. To measure match proximity, we calculated standardized mean difference (SMD) values and considered standardized mean difference (SMD) 0.2–0.5 as small, values of 0.5–0.8 as medium, and values > 0.8 as large [32].

### Corpus development

To create our corpus, EHR notes were extracted from the CDW from within 30 days before death for cases and matched end-date for controls. We chose to examine notes within 30 days of case death based on prior research about the importance of the interval immediately before death by suicide [20, 21]. We then removed notes between 0 and 5 days preceding date of case death to avoid potential endogeneity from incorrect entry of post-mortem data [33]. After restricting notes to 5–30 days prior to death, our final sample included 2842 cases and 14,042 controls. We chose not to exclude templated EHR, as the terms in these templates may offer predictive benefit.

### NLP methods

Our NLP methods utilized alternative analytical pipelines. For our semantic method, we used SÉANCE to analyze sentiment in clinical notes, transforming our corpus into 516 semantic variables. SÉANCE is a Python-based software package that is accessible on VA servers and has been found to be comparable to the commonly used Linguistic Inquiry and Word Count software [34]. SÉANCE utilizes a variety of established linguistic databases, including SemanticNet [35], General Inquirer Database [36], EmoLex [37], Lasswell [38], Valence Aware Dictionary and sEntiment Reasoner [39], Hu–Liu [40], Harvard IV-4 [36], and the Geneva Affect Label Coder [41]. Each SÉANCE database consists of expert-derived dictionary lists and rule-based systems [42], which can be assessed in positive and negative iterations. Examples include the Kin dictionary from Harvard IV-4 which consists of 50 terms denoting kinship, and the Pleasure dictionary from General Inquirer Database which consists of 168 words indicating the enjoyment of a feeling. For our count method, we first converted note corpora to lowercase, removed stop words like “his/hers”, “were/would”, “and/with”, and tokenized our data set into unigrams or bigrams. We then used the CountVectorizer module from the scikit-learn library [43] to convert corpus into a matrix of token counts. Within CountVectorizer, we set the min_df and max_df parameters to 100 and 0.9, respectively. This means that unigrams and bigrams that occurred in fewer than 100 documents or more than 90% of documents are not included as count features. This was done to ensure that neither exceedingly rare nor exceedingly common terms were included in our data set. The Resulting count-matrices and SÉANCE features were represented using numerical matrices consisting of a unique clinical note per row [44]. We also investigated using term frequency–inverse document frequency (TF-IDF) [45] instead of counts but found slightly better predictive performance for most risk tiers using counts. A summary table of results and hyperparameters for the joint matrix models using this NLP method are found in Supplementary Table 1.

### Data preprocessing

We created a joint matrix of count and semantic terms by horizontally merging the feature matrices from the SÉANCE and CountVectorizer modules by unique document number. We did this individually for each risk-tier, resulting in joint matrices with different number of columns since the word count feature matrix is dependent on the number of unique unigrams or bigrams in the corpus.

High, moderate, and low-risk samples were then randomly assigned into training (64% of sample), validation (16% of sample), and testing sets (20% of sample). We allocated notes belonging to the same patient to the same partition to prevent leakage of information between training, validation, and testing data. Missing values were retained, and data remained unnormalized due to the flexibility of decision tree approaches in handling this information [46].

To avoid potential over-selection of count variables relative to semantic variables, due to having far more count variables in the joint matrices than semantic variables, we weighted the selection probabilities for count and semantic features. These feature weights affect the probability of a certain feature set (e.g., semantic or count) being selected by the model for evaluation at the split of each node. If a feature weight of 1 was applied to all semantic and count variables, all variables would have equal probability of being selected at a node split. However, since there are far more count variables than semantic variables across all risk-tier joint matrices, the probability of a count variable being randomly selected from all available variables is higher than the probability of selecting a semantic variable from all given variables. By applying a varying feature weight to the semantic features, and a constant weight of 1 to all count variables, we could change the probability that a semantic variable would be chosen at a node split relative to the probability of a count variable being chosen.

The selection probability applied to the semantic features is proportional to the ratio of count features to semantic features times alpha ($\alpha$), a variable weighting parameter, as seen below. The feature weight applied to all count features were held constant at 1.

$${feature}\,{weights}=\left(\frac{{n}_{count}}{{n}_{semantic}}\right)* \alpha$$

The differing feature weights account for the differing number of count variables in the feature matrix but not to the probability of either type of variable to be selected by the model. The probability of any given semantic variable to be selected from the set of semantic variables that make up the feature matrix is summarized by β, a ratio of $\alpha$:

$${\rm{\beta }}=\frac{\alpha }{\alpha +1}$$

The values of $\alpha$ investigated were: 0.01, 0.03, 0.06, 0.10, 0.30, 0.60, 1.00, 2.50, 5.00. The results are presented as a function of β, where the equivalent β values were 0.01, 0.03, 0.06, 0.09, 0.23, 0.38, 0.50, 0.71, and 0.83. By changing the value of β incrementally, we were able to investigate change in model performance for each risk-tier as semantic features were increasingly more likely to be selected in comparison to count features. For the sake of brevity, only the best-performing of these models are presented and discussed in the body of this report. The performances of the remaining models are presented in Supplementary Table 2. We also generated models trained on just count features and just semantic features to compare to the performance of our models built on weighted joint features. In this way, we were able to evaluate whether incorporating feature weights in a model trained on both count and semantic variables would be able to comparatively to models trained on each data type separately.

### Model selection

To develop classification models, we used XGBoost [47] a decision-tree classifier that continually refines subsequent decision trees to correct potential errors from previous iterations in a process known as “boosting.” We selected XGBoost due to its computational efficiency and resistance to bias from collinearity, which are particular concerns for data sets such as ours, which are very large and may have many collinear features since unigrams or bigrams may frequently co-occur within a document or be included as terms within a SÉANCE variable [48]. Additionally, research indicates XGBoost often results in the highest area under the receiver operating characteristic curve (AUC), a common evaluation metric for binary classification, for suicide risk prediction models [49]. We have also previously found that XGBoost offered increased predictive accuracy relative to other algorithms in a related dataset [20]. Lastly, unlike other tree-based classifiers like Random Forest, XGBoost predictors can be assigned weights, enabling us to utilize this weighting approach to account for feature imbalance. We trained these models on the vectors of individual documents because training on note-level data rather than patient-level data resulted in a much larger sample size.

### Model evaluation

To evaluate the overall performance of each model, we calculated the patient-level AUC because our outcome (suicide) is on the patient-level. Although suicide risk prediction models have historically struggled with sub-clinical predictive performance [50], since our sample was matched based on REACH-VET risk, an AUC greater than 0.50 does not reflect the accuracy of our model over chance, but rather indicates improved predictive accuracy over the REACH-VET algorithm. Within this context, an AUC of 0.60, accordingly, infers that a random case has a 60% chance of having a higher predicted score compared to a random control, conditioning on matching REACH-VET score. We have also included other metrics, such as Brier score, accuracy, and precision in Table 1. To calculate the AUC, we determined patient-level probabilities by averaging probabilities at the note level from our withheld test set. We estimated 95% confidence intervals (CI) for our AUC’s via 1000-sample non-parametric bootstrapping. We used XGBoost (Version 2.0.3), and Scikit-learn (Version 0.23.1) to develop the models. The model and parameters from the grid search that resulted in the best AUC on a withheld validation set were applied to a withheld test set. A 100-iteration randomized grid search employing 5-fold cross-validation was used for coarse hyperparameter selection (see Supplementary Table 1 for list of hyperparameters). Searches were conducted for each risk-tier and feature weighting scheme applied to the semantic features. We considered the model with the highest AUC as the “best performing” model. We gauged model significance by evaluating confidence level overlap, such that if models’ confidence intervals did not overlap they were considered as being significantly different [51].

**Table 1:** This table presents the descriptive characteristics of Veterans Affairs (VA) patients who died by suicide in 2017 and 2018 (cases) and risk matched patients who did not die during those intervals (controls).

	Case	Control	SMD
	(N = 2842)	(N = 14042)	
**Risk-tier**			0.007
High	389 (13.7%)	1940 (13.8%)	
Moderate	1436 (50.5%)	7040 (50.2%)	
Low	1017 (35.8%)	5044 (36.0%)	
**Document Count**			
High (Mean (SD))	37.9 (53.9)	40.0 (82.3)	0.031
Moderate (Mean (SD))	15.0 (32.7)	12.2 (40.9)	0.076
Low (Mean (SD))	9.1 (18.5)	7.0 (19.0)	0.113
**Demographics**			
Female	119 (4.2%)	1079 (7.7%)	0.149
Non married	1688 (59.4%)	7861 (56.1%)	0.068
Married	1154 (40.6%)	6163 (43.9%)	0.038
Homeless_prior24m	212 (7.5%)	1189 (8.5%)	0.067
Veteran	2834 (99.7%)	13971 (99.6%)	0.017
Rural	635 (22.3%)	3215 (22.9%)	0.013
**Race**			0.273
Am_Ind or Asian_Pac	61 (2.1%)	308 (2.2%)	
Black	154 (5.4%)	1638 (11.7%)	
Hispanic	124 (4.4%)	875 (6.2%)	
Unknown	129 (4.5%)	306 (2.2%)	
White	2374 (83.5%)	10897 (77.7%)	
**Age**			0.008
Mean (SD)	60.5 (18.0)	60.4 (15.7)	
Median [Min, Max]	64.0 [21.0, 101]	63.0 [18.0, 101]	
**Deployment**			
Vietnam	1100 (38.7%)	5862 (41.8%)	0.066
Afghanistan or Iraq	957 (33.7%)	4761 (33.9%)	0.017
**Mental Health Diagnosis/ Risk Flag**			
Anxiety	1341 (47.2%)	6686 (47.7%)	0.009
Bipolar	545 (19.2%)	2238 (16.0%)	0.085
Conduct	56 (2.0%)	316 (2.3%)	0.020
Depression	1876 (66.0%)	9137 (65.2%)	0.020
Neurocognitive	316 (11.1%)	1671 (11.9%)	0.025
OCD	80 (2.8%)	325 (2.3%)	0.032
PTSD	1060 (37.3%)	5273 (37.6%)	0.005
Personality	389 (13.7%)	1599 (11.4%)	0.070
Sleeping	1331 (46.8%)	7270 (51.8%)	0.100
Substance	1249 (43.9%)	5401 (38.5%)	0.112
Trauma	1442 (50.7%)	7235 (51.6%)	0.016
Combat	731 (25.7%)	2680 (19.1%)	0.159
Military Sexual Trauma	126 (4.4%)	875 (6.2%)	0.080
**Number of Inpatient Mental Health Days within 1 Year of Death**			0.024
Mean (SD)	17.2 (66.1)	15.6 (64.6)	
Median [Min, Max]	0 [0, 366]	0 [0, 366]	
**Prescriptions**			
Opioid Rx_prior12	885 (31.1%)	4338 (30.9%)	0.004
Opioid Rx_prior24	1104 (38.8%)	5686 (40.5%)	0.035
Mood Stabilizer Rx_prior12	1017 (35.8%)	4718 (33.6%)	0.045
Mood Stabilizer Rx_prior24	1178 (41.4%)	5455 (38.9%)	0.052
Antipsychotic Rx_prior12	616 (21.7%)	2364 (16.9%)	0.122
Antipsychotic Rx_prior24	708 (24.9%)	2791 (19.9%)	0.120
Antidepressant Rx_prior12	1573 (55.3%)	7661 (54.6%)	0.014
Antidepressant Rx_prior24	1733 (61.0%)	8401 (59.9%)	0.022

Standardized mean difference (SMD) values evaluate the degree of match similarity. We considered standardized mean difference (SMD) 0.2-0.5 as small, values of 0.5-0.8 as medium, and values > 0.8 as large [32].

Variables marked as “Am_Ind or Asian_Pac” reference American Indians or Pacific Islanders.

Variables marked as “_prior12” include data from the 12 months before death. Variables marked as “_prior24” include data from the 24 months prior to death.

### Ethics approval and consent to participate

Our local institutional review board, the Veterans IRB of Northern New England, determined that informed consent was not needed under the 2018 Common Rule, given the study’s reliance on retrospective EHR data. This study, #1582333, is part of the project titled, “Leveraging Natural Language Processing to Evaluate Suicide Risk in Electronic Medical Record Notes”. All procedures comply with the ethical standards of the relevant national and institutional committees on human experimentation and with the Helsinki Declaration of 1975, as revised in 2008. Materials and analysis code for this study are not available due to the clinical nature of our study and federal protections for Veteran data.

## Results

### Sample

For the high-risk subgroup, we identified a set of 92,399 notes from 389 cases and 1940 controls. For the moderate-risk subgroup, we identified a set of 107,532 notes, from 1436 cases and 7040 controls. For the low-risk sub-group, we identified a set of 44,613 notes, from 1017 cases and 5044 controls. The demographics for the entire cohort are described in Table 2. Cases and controls had low standardized mean differences between all considered demographics. The variable with the highest SMD was race, where a larger proportion of controls identified as black compared to cases. Our group has previously identified and discussed this discrepancy as a limitation of REACH-VET [23]. For all risk tiers, the number of semantic variables included in the feature matrix was 516. The number of word count variables in the feature matrix was 25,242 for high risk, 27,479 for moderate risk, and 11,018 for low risk.

**Table 2:** This table presents additional model metrics for the best-performing natural language processing derived suicide risk prediction models for suicide risk stratified (high, moderate, low) sample of Veterans Affairs (VA) patients who died by suicide in 2017 and 2018 (cases) and risk matched patients who did not die during those intervals (controls).

Risk Tier	Metric	Semantic Model		Count Model		Joint Model	
		Document-Level	Patient-Level	Document-Level	Patient-Level	Document-Level	Patient-Level
High	Brier Score	0.1634	0.1867	0.1680	0.1867	0.1658	0.1867
	Accuracy	0.8011	0.8133	0.7898	0.8133	0.7918	0.8133
	Precision	0.2623	0.0000	0.1911	0.0000	0.1951	0.0000
	Recall	0.0044	0.0000	0.0201	0.0000	0.0176	0.0000
	Specificity	0.9970	1.0000	0.9791	1.0000	0.9821	1.0000
	PPV	0.2623	–	0.1911	–	0.1951	–
	NPV	0.8028	0.8133	0.8025	0.8133	0.8026	0.8133
	F1	0.0087	0.0000	0.0364	0.0000	0.0323	0.0000
Moderate	Brier Score	0.1792	0.1669	0.1793	0.1668	0.1808	0.1680
	Accuracy	0.7677	0.8331	0.7647	0.8302	0.7617	0.8320
	Precision	0.2575	0.0000	0.3945	0.0000	0.3547	0.1429
	Recall	0.0145	0.0000	0.0758	0.0000	0.0663	0.0036
	Specificity	0.9878	0.9979	0.9660	0.9979	0.9647	0.9958
	PPV	0.2575	0.0000	0.3945	0.1667	0.3547	0.1429
	NPV	0.7743	0.8346	0.7815	0.8349	0.7796	0.8348
	F1	0.0274	0.0000	0.1271	0.0000	0.1117	0.0070
Low	Brier Score	0.1697	0.1756	0.1602	0.1739	0.1789	0.1805
	Accuracy	0.7829	0.8244	0.7928	0.8261	0.7836	0.8195
	Precision	0.3547	0.5000	0.4684	0.7500	0.1493	0.0000
	Recall	0.0692	0.0282	0.0629	0.0141	0.0113	0.0000
	PPV	0.3547	0.5000	0.4684	0.7500	0.1493	0.0000
	NPV	0.8008	0.8276	0.8020	0.8263	0.7937	0.8235
	Specificity	0.9675	0.9940	0.9815	0.9990	0.9833	0.9940
	F1	0.1157	0.0533	0.1109	0.0276	0.0211	0.0000

These predictive models were developed for each risk-tier using count vectorized matrix, the joint count-semantic matrix, and the semantic matrix methods. Models were developed using XGBoost [47].

### Risk variables

Derived count and semantic risk variables emphasized discrete risk domains. The high-risk count model included terms related to acute psychiatric concerns (“suicide”, “anxiety”, “alcohol”), moderate-risk count model included terms related to structured care processes (“management”, “health”, “objective”), and the low-risk count model included terms related to medical and rehabilitative terminology (“dementia”, “chemotherapy”, and “ICU”). The high-risk semantic model included variables related to negativity and emotional distress (“Valence_neg_3”, a variable connected to negative emotions, “Submit_GI”, a variable connected to submission and passivity, and “Object_GI_neg_3”, a variable related to negative object references), the moderate-risk semantic model included variables related to lack of emotional engagement (“Sensitivity_neg_3”, a variable connected to lack of sensitivity, “Solve_GI_neg_3 ”, a variable connected to lack of problem solving ability, and “Powoth_Lasswell_neg_3”, a variable connected to lack of power over other people), and the low-risk semantic model included variables related to social connection (“Positive_Emolex”, a variable connected to positive emotion, “Exert_GI)”, a variable connected to effort and energy, and “Socrel_GI”, a variable connected to social relationships). The top 25 most important variables per model are listed in Supplementary Table 3a–c.

### Models

To confirm our matching methodology and the functional ability of our derived models to offer predictive benefit over and above REACH-VET, we ran a prediction model exclusively using the REACH-VET model. As expected, this model yielded an AUC of 0.50, indicating that cases and controls were closely matched and that subsequent derived prediction models which yielded AUC values over and above this benchmark could offer enhanced predictive relative to REACH-VET.

For the high-risk-tier, although no model performed significantly better than any other, the count model offered the highest predictive performance. For moderate-risk and low-risk-tiers, the semantic models offered significantly or almost significantly worse predictive performance when compared to the count or joint models. Across all risk-tiers, count-based models consistently outperformed semantic models, as noted in Table 3. For moderate and low-risk-tier joint models, the probability of selecting semantic variables, β, was relatively low (0.03), indicating the strong role of count variables in achieving high predictive performance in these subgroups. Our high-risk-tier joint model, though it achieved only comparable performance to the count model, had a β of 0.38 - one of the higher β values we investigated - indicating a greater emphasis on semantic variables in a joint context. As all models achieved AUC’s greater than 0.50, they offered predictive improvements over and above REACH-VET’s predictive accuracy. Our best joint models had AUC’s of 0.66 (for moderate risk tier) and 0.69 (for low risk tier) representing moderate incremental discrimination for the moderate- and low-risk patient groups over and above REACH-VET’s predictive benchmark. (See Supplementary Figure 1 for all model prediction curves, including initial REACH-VET model).

**Table 3:** This table presents natural language processing derived suicide risk prediction models for suicide risk stratified (high, moderate, low) sample of Veterans Affairs (VA) patients who died by suicide in 2017 and 2018 (cases) and risk matched patients who did not die during those intervals (controls).

Risk-tier	Semantic Model AUC [95% CI]	Count Model AUC [95% CI]	Joint Model AUC [95% CI]	Beta^a^
High	0.56 [0.49–0.63]	0.62 [0.54–0.68]	0.61 [0.51–0.65]	0.38
Moderate	0.59 [0.55–0.63]	0.65 [0.61–0.68]	0.66 [0.62–0.69]	0.03
Low	0.60 [0.55–0.63]	0.67 [0.63–0.71]	0.69 [0.64–0.72]	0.03

The table identifies the best-performing predictive models for each risk-tier using count vectorized matrix, the joint count-semantic matrix, and the semantic matrix methods. Models were developed using XGBoost [47]. Predictive performance was measured by area under the ROC curve (AUC). Beta describes the optimal weighting between semantic and count variables in the joint model. Since our sample was matched on REACH-VET risk, an AUC greater than 0.50 indicates improved predictive accuracy over the REACH-VET algorithm. An AUC of 0.69 thus infers that a random case has a 69% chance of having a higher predicted score compared to a random control, conditioning on matching REACH-VET score.

^a^Applied in case of Optimal Joint Model.

Across all models, accuracy improved as risk decreased from high-risk to low-risk. For high-risk-tier patients, even though the count-only model showed the best overall performance, the top features in the joint model tended to be semantic, as indicated by the β being 0.38, meaning the probability of selecting a semantic variable is higher than the probability of selecting a count variable. Notably, despite the very different β values for the best performing joint models in each tier, the most important variables within each model were a blend both semantic and count-based elements, suggesting their synergistic potential.

## Discussion

In this study, we evaluated alternative methods of leveraging NLP-transformed unstructured EHR data in a suicide risk-tier stratified sample. We developed a weighting strategy that optimally incorporated both semantic risk variables from a “closed vocabulary” approach and count-based variables from an “open vocabulary” approach to predict suicide risk across sample risk-tiers. Our findings highlight inherent differences in each group’s corpora, reiterating that each risk-tier is demographically distinct and received very different care.

The best performing high-risk joint model had an elevated β, indicating that in this context, semantic variables were influential in achieving an AUC comparable to the models built on just one variable type. The lack of significant differences in model performance for this risk-tier may indicate that in subpopulations with more EHR text documents, and therefore larger corpus vocabularies, less is gained by leveraging multiple NLP methods [52, 53]. It is also likely that predictive metrics like REACH-VET that are specifically developed for high-risk classification already include refined risk variables to target the high-risk population and thus already offer enhanced benefits. Additional NLP-derived personalized risk variables may, accordingly, offer decreased benefit for the high-risk population. In contrast, moderate-risk and low-risk populations, who are not particularly prioritized in current predictive risk modeling methods, stand to benefit more from increased utilization of NLP-derived personalized risk variables.

For moderate-risk and low-risk patients, the joint model offered somewhat higher predictive performance than the other models. Both best-performing joint models had a β of 0.03, which was one of the lowest β values we investigated. This weighting suggests that semantic variables, relative to the count variables, were less influential in achieving optimal predictive performance. As the moderate and low-risk-tier patients tend to receive less care, and accordingly have less EHR notes, when compared to high-risk patients, it is likely that they have smaller linguistic vocabularies [23]. Having smaller vocabularies may reduce the relevance of derived semantic variables [26] and limit the benefit of leveraging these variables in classification models. Count models, in contrast, do not require extensive vocabularies and might offer increased benefits for moderate-risk and low-risk patient groups.

Additional research into interactions between variables in the joint matrix for each risk-tier may shed light into why the influence of semantic variables on predictive performance, relative to count variables. In contrast to previous studies that emphasized the value of exclusively utilizing closed vocabulary approaches when building classification models for psychological applications [34, 36], our findings suggest that semantic analysis’ predictive value can increase when leveraged alongside open vocabulary approaches such as word count. Our method provides a novel mechanism to optimally include both approaches in the same model, tailored to each subgroup’s unique risk criterion. More broadly, including risk variables derived from unstructured EHR may offer additional predictive improvement for non-high-risk populations as these groups typically have fewer medical encounters and less structured EHR, data formats that are key to REACH-VET and related predictive models [54].

### Methodological implications

This study demonstrates the feasibility and efficacy of incorporating data from two discrete NLP methods into a singular model to enhance population-specific suicide risk classification. These findings expand on our research showing that supplementing conventional risk factors with nuanced psychosocial insights pulled from previously unusable unstructured EHR notes can improve accuracy and risk assessment capabilities. As evidenced by the moderate- and low-risk groups’ elevated joint model AUC scores, our methodology affirms the benefits of utilizing multimodal NLP methods in particular for patients with sparse structured data, a trend noted in recent publications in other research domains [55–58]. It is of note that the low-risk group, the group with the fewest clinical encounters, benefited the most from our multimodal approach, experiencing the highest level of predictive gain over relative to REACH-VET.

Our study illustrates how a classification model can be built on a matrix of different types of structured or NLP transformed data by accounting for feature weight is incorporated to account within the model joint matrix. This method could be used to include feature interactions from disparate data formats, for example, demographics and sentiment variables. By mining for interactions, we can better understand the underlying populations and tailor future classification and predictive models for more accurate identification of at-risk patients. These findings offer a promising step towards targeted suicide prevention efforts within the VA healthcare system.

### Clinical implications

Whereas most VA suicide prediction and prevention services pragmatically center on patients with the highest classified risk [5], individuals who tend to access a variety of mental health services and epidemiologically are widely studied [59], low-risk patients are frequently underserved and poorly epidemiologically understood [6, 23]. Indeed, these patients’ low-risk status references current suicide risk classification techniques’ inability to accurately conceptualize their actual risk status. Our own previous research has shown that lower risk patients utilize VA resources less often and thus have less structured data for the algorithm to learn on [23]. Having more data available for REACH-VET has shown to improve the predictive accuracy of the model [6]. By better identifying these patients and enhancing classification methodologies, our derived method could help improve understanding of risk typologies and prediction strategies.

Effective identification of individual-level suicide risk variable constellations could aid the development of tailored risk-tier specific suicide prevention interventions, a priority championed by public health approaches to suicide prevention [60, 61]. Even with the successfully development of suicide prediction and prevention interventions for patients with the highest suicide risk [59, 61], a recent special Lancet issue about suicide and public health suggests that “there will never be enough adequately trained mental health professionals to deliver one-on-one treatment to suicidal individuals…” [60] Scholars argue instead that targeted interventions which integrate individual-level risk variables and social determinants of health will allow more effective suicide prevention services, especially for patients at lower-risk tiers[60, 62, 63]. Interventions for lower-risk populations could include automated symptom check-ins, referral to peer support or wellness programs, incorporating psychosocial risk factors into routine clinical conversations, or linking patients to general health interventions that promote protective factors such as sleep, exercise, or social engagement. These lower-intensity or indirect strategies—if guided by improved risk stratification—may help reduce risk in its early stages and support prevention efforts before more acute interventions become necessary. We are hopeful that our methodology advances identification of these target populations and their unique risk and treatment domains.

It is of note that incorporating semantic variables into the moderate- and low-risk groups allowed enhanced evaluation of patients’ psychosocial domains, including appraisal of emotional engagement for moderate-risk and social connection for low-risk patients. These characteristics reference the centrality of interpersonal suicide risk domains [64] and provide a scalable method to assesses related risk criteria.

### Limitations

We used a retrospective sample because it was the most appropriate format for studying suicide deaths among VA patients. As we only used VA data, the importance of specific features we found are not necessarily generalizable to the larger population. However, the weighting method we present to combine different NLP modalities is not specific to the Veteran context and we holds promise for broader usage.

When it comes to the EHR notes themselves, there are a few limitations. Firstly, we have limited access to EHR notes from our VA patients’ non-VA medical providers, which may contribute to the prevalent lack of documentation among the lower risk-tiers, a population that may use VA services less than those at higher predicted risk-tier [65]. As the documents in our study were written by providers about their patients, rather than by patients about themselves, our findings may be constrained by providers’ personal biases [66]. This concern may attenuate the effects of the risk factors and could constrain subsequent clinical engagement around these issues. We also chose to leave clinical acronyms such as “SI” for “suicidal ideation” in the text. These features may occur in similar documents or even co-occur, making them collinear. The design of XGBoost makes it almost immune to multicollinearity, but it may also be true that in the case of clinical acronyms, if we had completely replaced acronyms with the full phrases they represent, the terms of the full phrases could have higher reported importances overall. Additionally, ambiguous acronyms such as “SA” for “suicide attempt”, “substance abuse”, or “sexual assault/abuse” could be assigned incorrect meaning during post-hoc interpretation. We chose to utilize a count-based NLP approach as it is relatively computationally inexpensive, however, one limitation of this approach is that it captures little context, aside from term frequency, which may limit the predictive nature of the resulting variables. Finally, we restricted to 5-30 days of EHR notes based on our previous research, but a larger or smaller inclusion window could lead to different predictive performance [20, 21].

There are also a few limitations relating to model development. Although we were able to achieve significantly higher predictive accuracy than REACH-VET with most of our XGBoost models, alternative analytical tools, data-processing methods, or other model classes, such as regularized logistic regression, may offer even better performance. We plan to conduct these comparisons in future work. By including so many variables in the model and weighting features rather than conducting dimensionality reduction prior to modelling, we run the risk of overfitting. Future work will also seek to validate this methodology in the light of this limitation.

Lastly, there are a few limitations regarding our design. Our use of a matched case-control design may introduce temporal bias because our matched controls may not have the same risk profile as a random sample [67]. We have however, attempted to reduce the effects of temporal bias by (1) not including any documents within 5 days of death since some of these documents may be post-mortem notes if date of death is incorrect and (2) pulling notes from equivalent time periods for both cases and controls to ensure “follow-up” time is identical. Secondly, our method offered less benefit for high-risk patients, in prior studies we found that risk modeling for this population can be enhanced by using alternative machine learning methods [20]. Given this group’s increased service usage relative to the other patient groups [23], it is likely that more minute sub-risk tier characterization [6], along with methods which optimally weight structured and unstructured data, including demographics [68], will offer particular benefit for this population. Our models were developed and evaluated on retrospective data and thus may not maintain the same performance if applied prospectively. Future work would be needed to determine real-world performance.

As our study was primarily intended to refine a novel methodology, it did not target broader operationalization. As we developed it exclusively using VA data, VA research servers, and VA approved software, it could be refined for implementation or incorporated within future REACH-VET rollouts. Whereas more sophisticated machine learning methods require substantial computational resources [69], which may exceed currently allocated VA processing capabilities, our derived method is comparatively simple and can be completed using existing computational resources. Additionally, the high-dimensional inputs and non-linear interactions of more complex machine learning methods, such as deep learning, make isolating model decisions and determining the clinical impact of these models difficult [70, 71]. In contrast, our method’s reliance on comparatively simple format variables, including pre-established psychosocial risk variables and count-based variables allows more straightforward interpretation [26, 34]. In the future, we look forward to partnering with clinical investigators to evaluate the potential clinical relevance of derived models. Future research will focus on further increasing risk personalization via incorporating service utilization, medication usage, evidence-based care, and availability of psychosocial care, interactions between structured and unstructured data formats, and risk changes over time. As VA computational resources expand, we also plan to implement deep learning analytic pipelines involving transformers, large language models, and neural nets, such as those employed in related suicide attempt risk analyses [72].

## Supplementary information

Supplementary Tables and Figures

## Data Availability

Data access is restricted due to the clinical nature of dataset and VA privacy protections.
