# Supplementary material for: Enhancing personalized suicide risk prediction for VA patients by integrating discrete natural language processing models
Source: Transl Psychiatry. 2026 Mar 20;16:196. doi: 10.1038/s41398-026-03940-8 (PMC13039941; doi:10.1038/s41398-026-03940-8)
Supplement: Supplementary file 1 — Supplementary Tables and Figures [file 41398_2026_3940_MOESM1_ESM.docx]

**Supplemental Figure 1**

*Receiver operating curve (ROC) for the best-performing multimodal XGBoost model for each tier of predicted suicide risk in our risk-matched cohort. Each curve is created by plotting the proportion of cases correctly identified by the model as cases over the proportions of controls incorrectly identified by the model as cases. The predictive performance of the model can be summarized by the area under the curve (AUC). The AUC of high, moderate, and low risk models were 0.61, 0.66, and 0.69, respectively. See Table 2 for 95% confidence intervals. Because our cohort is matched on predicted suicide risk, a classification model of predicted suicide risk alone performs no better than chance. The ROC of this null model, including patients from all suicide risk tiers, is plotted as a dotted line and the AUC is 0.5.*


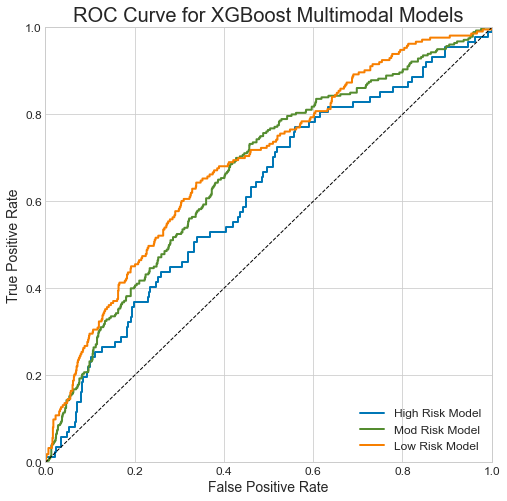


**Supplementary Table 1.** This table presents natural language processing derived suicide risk prediction models for suicide risk stratified (high, moderate, low) sample of Veterans Affairs (VA) patients who died by suicide in 2017 and 2018 (cases) and risk matched patients who did not die during those intervals (controls). The table identifies the final hyperparameters for each XGBoost (Chen and Guestrin, 2016) model that had the highest area under the receiver operating curve (AUC) when evaluated on a withheld test set.

| **Risk Tier** | **Parameters** | **Count Model** | **Optimal Joint Model** | **Semantic Model** |
| --- | --- | --- | --- | --- |
| High | colsample_bynode | 1 | 0.75 | 0.75 |
|  | colsample_bylevel | 0.25 | 1 | 0.75 |
|  | colsample_bytree | 0.75 | 0.25 | 1.0 |
|  | min_child_weight | 10 | 5 | 1 |
|  | max_depth | 5 | 4 | 3 |
|  | gamma | 1 | 1.5 | 0.5 |
|  | num_boost_round | 200 | 100 | 100 |
| Moderate | colsample_bynode | 0.5 | 0.25 | 0.75 |
|  | colsample_bylevel | 0.5 | 1 | 0.75 |
|  | colsample_bytree | 0.5 | 0.25 | 0.75 |
|  | min_child_weight | 1 | 5 | 1 |
|  | max_depth | 5 | 4 | 4 |
|  | gamma | 2 | 1.5 | 1 |
|  | num_boost_round | 200 | 300 | 100 |
| Low | colsample_bynode | 0.75 | 0.5 | 0.5 |
|  | colsample_bylevel | 0.75 | 0.25 | 0.75 |
|  | colsample_bytree | 0.75 | 0.25 | 0.5 |
|  | min_child_weight | 5 | 5 | 5 |
|  | max_depth | 4 | 4 | 5 |
|  | gamma | 5 | 5 | 1.5 |
|  | num_boost_round | 100 | 200 | 200 |

*Notes.* colsample_bynode is the subsample ratio of columns for each node; colsample_bylevel is the subsample ratio of columns for each level; colsample_bytree is the subsample ratio of columns when constructing each tree; min_child_weight is the minimum sum of instance weight; max_depth is the maximum depth of a tree; gamma is a mean of gamma distribution; num_boost_round is number of boosting rounds or trees.

**Supplementary Table 2**

*This table presents natural language processing derived suicide risk prediction models for suicide risk stratified (high, moderate, low) sample of Veterans Affairs (VA) patients who died by suicide in 2017 and 2018 (cases) and risk matched patients who did not die during those intervals (controls). The table compares alternative Beta weights, indicating the probability of the model selecting a semantic variable relative to a count variable from the feature matrix, for high, moderate, and low-suicide-risk patients. Predictive performance on a withheld test set, as evaluated by area under the receiver operating curve (AUC), is presented for each model. As cases and controls were matched on the VA’s leading suicide risk prediction metric, an AUC > 0.50 represents a predictive increase over and above this metric.*

| **Beta** | **High** | **Moderate** | **Low** |
| --- | --- | --- | --- |
| 0.01 | 0.587 | 0.656 | 0.667 |
| 0.03 | 0.606 | 0.659 | 0.687 |
| 0.06 | 0.594 | 0.64 | 0.659 |
| 0.09 | 0.578 | 0.645 | 0.682 |
| 0.23 | 0.596 | 0.657 | 0.666 |
| 0.38 | 0.611 | 0.642 | 0.678 |
| 0.50 | 0.582 | 0.642 | 0.667 |
| 0.71 | 0.582 | 0.647 | 0.678 |
| 0.83 | 0.582 | 0.647 | 0.678 |

**Supplementary Table 3a.** Top 25 most important variables, in descending order, from each high-risk XGBoost (Chen and Guestrin, 2016) model presented in Table 2. The Count Model only presents linguistic terms. The Optimal Joint Model presents mix of terms and semantic variables. The Semantic Model only presents semantic variables. Semantic variables were derived using SÉANCE (Crossley et al., 2017). In the Optimal Joint Model and Semantic Model columns, we include the relevant Psychosocial Construct as well as the original SÉANCE Variable. For example, Natural Processes is a Psychosocial Construct that is identified by the SÉANCE variable, Natrpro_GI. SÉANCE variables include both positive and negative versions of each semantic variables, based on if negated terms are included within the model. Negative versions are indicated by “_neg_3”.

| **Count Model** | **Optimal Joint Model** | **Semantic Model** |
| --- | --- | --- |
| *Term* | *Psychosocial Construct  (SÉANCE Variable) or Term* | *Psychosocial Construct  (SÉANCE Variable)* |
| Patient | Work (Work_GI) | Valence (Valence_neg_3) |
| Care | Mental/Emotional states (Sv_GI) | Skill (Sklpt_Lasswell_neg_3) |
| Discharge | Submission (Submit_GI_neg_3) | Negative Adjectives (negative_adjectives_component) |
| Use | Failure (Fail_GI_neg_3) | Polarity Nouns (polarity_nouns_component) |
| Alcohol | Wellbeing Gain (Wlbgain_Lasswell_neg_3) | Emotion (Emot_GI) |
| Treatment | Fear (Fear_EmoLex) | Quality (Quality_GI_neg_3) |
| Medication | Compound (Vader_compound) | Action Verbs (Dav_GI_neg_3) |
| Anxiety | Submission (Submit_GI) | Power Total (Powtot_Lasswell_neg_3) |
| Suicide | Feelings (Feel_GI) | Location (Arenalw_Lasswell_neg_3) |
| About | Natural Processes (Natrpro_GI) | Virtue (virtue_adverbs_component) |
| Admission | Exertion (Exert_GI) | Fear (Fear_Emolex) |
| Call | Know (Know_GI) | Valence Valence (Valence) |
| Current | Negation (Negate_GI) | Arousal (Arousal) |
| Daughter | Settings (Arenalw_Lasswell) | Pleasantness (Pleasantness) |
| Day | Actors (Ptlw_Lasswell) | Attention (Attention) |
| Goal | Denial (Notlw_Lasswell) | Negative (vader_negative) |
| Group | Time Space (Timespc_Lasswell | Neutral (vader_neutral) |
| Mental | Contentment (Contentment_GALC_neg_3) | Submission (Submit_GI) |
| Need | Interest (Interest/Enthusiasm_GALC_neg_3) | Vice (Vice_GI) |
| Pain | Quantity (Quan_GI_neg_3) | Goal (Goal_GI) |
| Plan | Enlightenment (Endslw_Lasswell_neg_3) | Exertion (Exert_GI) |
| Problem | Certainty (certainty_component) | Know (Know_GI) |
| Active | Electroconvulsive Therapy | Abstraction (Abs_GI) |
| Assessment | Electroconvulsive | Quality (Quality_GI) |
| But | Monitor Assist | Power Loss (Powloss_Lasswell) |

**Supplementary Table 3b.** Top 25 most important variables, in descending order, from each moderate-risk XGBoost (Chen and Guestrin, 2016) model presented in Table 2. The Count Model only presents linguistic terms. The Optimal Joint Model presents mix of terms and semantic variables. The Semantic Model only presents semantic variables. Semantic variables were derived using SÉANCE (Crossley et al., 2017). In the Optimal Joint Model and Semantic Model columns, we include the relevant Psychosocial Construct as well as the original SÉANCE Variable. For example, Natural Processes is a Psychosocial Construct that is identified by the SÉANCE variable, Natrpro_GI. SÉANCE variables include both positive and negative versions of each semantic variables, based on if negated terms are included within the model. Negative versions are indicated by “_neg_3”.

| **Count Model** | **Optimal Joint Model** | **Semantic Model** |
| --- | --- | --- |
| *Term* | *Psychosocial Construct  (SÉANCE Variable) or Term* | *Psychosocial Construct  (SÉANCE Variable)* |
| Pain | Work (Work_GI) | Submission (Submit_GI) |
| Day | State Verbs (Sv_GI) | Doctrine (Doctrin_GI_neg_3) |
| Month | Submission (Submit_GI_neg_3) | Neutral (vader_neutral) |
| Plan | Failure (Fail_GI_neg_3) | Actors (Ptlw_Lasswell) |
| Active | Wellbeing Gain (Wlbgain_Lasswell_neg_3) | Failure (failure_component) |
| All | Fear (Fear_EmoLex) | Politics (Polit_GI) |
| Assessment | Vader_compound | Communication (Comform_GI) |
| Been | Submission (Submit_GI) | Know (Know_GI_neg_3) |
| Daily | Feelings (Feel_GI) | Arousal (Arousal) |
| Every | Natural Processes (Natrpro_GI) | Work (Work_GI) |
| Group | Exertion (Exert_GI) | Land (Land_GI) |
| Mouth | Know (Know_GI) | Wellbeing Total (Wlbto_Lasswell) |
| Needed | Negation (Negate_GI) | Sensitivity (sensitivity_neg_3) |
| Date | Locations (Arenalw_Lasswell) | Work (Work_GI_neg_3) |
| Health | Actors (Ptlw_Lasswell) | Solve (Solve_GI_neg_3 |
| HR | Denial (Notlw_Lasswell) | Power (Powoth_Lasswell_neg_3) |
| Note | Time Space (Timespc_Lasswell) | Objects (objects_component) |
| Patient | Contentment (Contentment_GALC_neg_3) | Positive (Positive_Emolex) |
| Given | Interest (Interest/Enthusiasm_GALC_neg_3) | Attention (Attention) |
| Management | Quantity (Quan_GI_neg_3) | Composite (vader_compound) |
| Medical | Enlightenment (Endslw_Lasswell_neg_3) | Economics (Econ_2_GI) |
| Medication | Certainty (certainty_component) | Social Relations (Socrel_GI) |
| Objective | Electroconvulsive Therapy | Male (Male_GI) |
| Outcome | Electroconvulsive | Trying (Try_GI) |
| Phone | Monitor assist | Persisting (Persist_GI) |

**Notes.** *Abbreviations, in alphabetical order.* HR; Human Relations.

**Supplementary Table 3c.** Top 25 most important variables, in descending order, from each low-risk XGBoost(Chen and Guestrin, 2016) model presented in Table 2. The Count Model only presents linguistic terms. The Optimal Joint Model presents mix of terms and semantic variables. The Semantic Model only presents semantic variables. Semantic variables were derived using SÉANCE (Crossley et al., 2017). In the Optimal Joint Model and Semantic Model columns, we include the relevant Psychosocial Construct as well as the original SÉANCE Variable. For example, Natural Processes is a Psychosocial Construct that is identified by the SÉANCE variable, Natrpro_GI. SÉANCE variables include both positive and negative versions of each semantic variables, based on if negated terms are included within the model. Negative versions are indicated by “_neg_3”.

| **Count Model** | *Psychosocial Construct  (SÉANCE Variable) or Term* | *Psychosocial Construct  (SÉANCE Variable)* |
| --- | --- | --- |
| *Term* | *Construct (SÉANCE Variable)  or Term* | *Construct (SÉANCE variable) or Term* |
| Client | Client | Object_GI_neg_3 |
| Dementia | PRRC | Polarity Verbs (polarity_verbs_component |
| Knee | Natural Processes (Natrpro_GI) Natrpro_GI | Negativity (vader_negative) |
| Last | Virtue (Virtue_GI_neg_3) | Valence (Valence) |
| Pain | Object (Object_GI_neg_3) | Social Order (social_order_component) |
| Patient | Continue | Joy (joy_component) |
| PRRC | Methadone | Economics (Econ_GI_neg_3) |
| Risk | PTRP | Expressivity (Exprsv_GI_neg_3) |
| Use | Hostile (Hostile_GI) | Interpretation of Action (Iav_GI_neg_3) |
| Area | Legal (Legal_GI) | Aptitude (Aptitude) |
| Attached Scanned | Negative Affect (Negaff_Lasswell) | Positivity (vader_positive) |
| Chemo | Affiliation (Affil_GI_neg_3) | Objects (Comnobj_GI) |
| Day | Objects (objects_component) | Communication (Comform_GI) Comform_I |
| Disorder | Area Every | Enlightenment (Endslw_Lasswell) |
| DOE | Bedtime Glaucoma | Joy (Joy_EmoLex_neg_3) |
| History | CABG | Work (Work_GI_neg_3) |
| Hyponatremia | Chemo | Space (Space_GI_neg_3) |
| ICU | Consciousness Alert | Affect (affect_friends_and_family_component) |
| Lab | David | Trust (trust_verbs_component) |
| Level | Dementia | Objects (objects_component) |
| Medical | Hyponatremia | Positivty (Positive_Emolex) |
| Medication | ICU | Strong (Strong_GI) |
| Methadone | Immunization | Interpretation of Action (Iav_GI) |
| Output Urine | QID PRN | Arousal (Arousal_nwords_neg_3) |
| Participated | Resident | Role (Role_GI_neg_3) |

**Notes.** *Abbreviations, in alphabetical order.* CABG; Coronary Artery Bypass Grafting; DOE: Department of Education; ICU: Intensive Care Unit; PRRC: Psychosocial Rehabilitation and Recovery Center; PTRP: Polytrauma Transitional Rehabilitation Program; QID PRN: Quater In Die (4x per day) Pro Re Nata (as needed).

**References**

Chen, T., Guestrin, C., 2016. XGBoost: A Scalable Tree Boosting System, in: Proceedings of the 22nd ACM SIGKDD International Conference on Knowledge Discovery and Data Mining. Presented at the KDD ’16: The 22nd ACM SIGKDD International Conference on Knowledge Discovery and Data Mining, ACM, San Francisco California USA, pp. 785–794. https://doi.org/10.1145/2939672.2939785

Crossley, S.A., Kyle, K., McNamara, D.S., 2017. Sentiment Analysis and Social Cognition Engine (SEANCE): An automatic tool for sentiment, social cognition, and social-order analysis. Behavior Research Methods 49, 803–821. https://doi.org/10.3758/s13428-016-0743-z
